# The Influence of Milling and Spark Plasma Sintering on the Microstructure and Properties of the Al7075 Alloy

**DOI:** 10.3390/ma11040547

**Published:** 2018-04-03

**Authors:** Orsolya Molnárová, Přemysl Málek, Jozef Veselý, Peter Minárik, František Lukáč, Tomáš Chráska, Pavel Novák, Filip Průša

**Affiliations:** 1 Department of Physics of Materials, Faculty of Mathematics and Physics, Charles University, Ke Karlovu 5, 12116 Prague, Czech Republic; malek@met.mff.cuni.cz (P.M.); vesely@gjh.sk (J.V.); peter.minarikk@gmail.com (P.M.); 2 Institute of Plasma Physics of the CAS, Za Slovankou 1782/3, 18200 Prague, Czech Republic; lukac@ipp.cas.cz (F.L.); chraskat@ipp.cas.cz (T.C.); 3 Department of Metals and Corrosion Engineering, UCT Prague, Technická 5, 16628 Prague, Czech Republic; Paja.Novak@vscht.cz (P.N.); Filip.Prusa@vscht.cz (F.P.)

**Keywords:** gas atomized Al7075 alloy, mechanical milling, spark plasma sintering, microstructure, microhardness

## Abstract

The compact samples of an Al7075 alloy were prepared by a combination of gas atomization, high energy milling, and spark plasma sintering. The predominantly cellular morphology observed in gas atomized powder particles was completely changed by mechanical milling. The continuous-like intermetallic phases present along intercellular boundaries were destroyed; nevertheless, a small amount of Mg(Zn,Cu,Al)_2_ phase was observed also in the milled powder. Milling resulted in a severe plastic deformation of the material and led to a reduction of grain size from several µm into the nanocrystalline region. The combination of these microstructural characteristics resulted in abnormally high microhardness values exceeding 300 HV. Consolidation through spark plasma sintering (SPS) resulted in bulk samples with negligible porosity. The heat exposition during SPS led to precipitation of intermetallic phases from the non-equilibrium microstructure of both gas atomized and milled powders. SPS of the milled powder resulted in a recrystallization of the severely deformed structure. An ultra-fine grained structure (grain size close to 500 nm) with grains divided primarily by high-angle boundaries was formed. A simultaneous release of stored deformation energy and an increase in the grain size caused a drop of microhardness to values close to 150 HV. This value was retained even after annealing at 425 °C.

## 1. Introduction

The commercial Al7075 alloy is a typical representative of age-hardenable aluminum alloys which derive their high strength especially from a high volume fraction of fine, homogeneously distributed strengthening particles (predominantly of GP zones and η′ phase) [1]. The formation of such phase composition and distribution requires a special thermal treatment. The strength can be additionally enhanced through a grain size refinement according to the Hall-Petch relation. The minimum grain size achievable in a conventional thermo-mechanically treated ingot metallurgical material is close to 10 µm [2]. Much finer grain sizes can be achieved using methods of severe plastic deformation [3]. Using six passes of equal channel angular pressing, grain size in a sub-micrometer range can be prepared [4]. The main drawback of such materials is their poor high temperature stability. Coarsening of the strengthening particles starts already at temperatures above 150 °C, the grain coarsening in the ECAP material was observed after annealing at 300 °C for 1 h [4,5]. Powder metallurgical processing route was therefore alternatively tested with the aim to prepare a fine-grained Al7075 alloy with improved microstructural stability at elevated temperatures.

High cooling rates used in the gas atomization process (10^3^–10^5^ K/s) along with a high surface to volume ratio of droplets lead to a fine microstructure, extended solid solubility, and formation of metastable phases [6], all enhancing the material’s strength. Further microstructural refinement can be achieved by mechanical milling of atomized powders. A high dislocation density is introduced into the powder particles during the milling process. Self-organization of dislocations into cell networks, low angle boundaries, and finally high angle boundaries are considered to be the mechanism of grain refinement down to the nanoscale range [7]. Further, milling can lead to the dissolution of primary phases and to the extension of solid solubility. This is very convenient for the precipitation of fine, homogeneously distributed second phase particles during the powder’s consolidation, which would lead to an increased strength of the final material. Additionally, fine oxide and nitride particles originating from the powder particle surface, atmosphere, or milling agent can be introduced into the matrix during milling, which can further enhance strength through dispersion strengthening [8]. These dispersoids also can play an important role in maintaining the powder’s fine-grained structure during the subsequent consolidation process.

Powder consolidation is probably the most serious problem of powder metallurgy as it requires generally high temperatures and long periods of sintering. During this process, the material tends to reach its thermodynamic equilibrium and many gains from atomization or milling can be lost (coarsening of the strengthening particles, reduction in dislocation density, grain growth). The spark plasma sintering/field assisted sintering technology (SPS/FAST) should minimize these undesirable processes [9]. This technique combines applied uniaxial pressure with heating by low voltage pulsed direct current (DC) flowing through the sample. High current density and large joule heat can be evolved at contact points of powder particles where the sintering occurs, whereas the powder particle interiors remain nearly unaffected and preserve their original microstructure. The existence of spark discharge linked to contact points is widely debated in literature. Some authors believe that it can break up the oxide layers present at the powder particle surface and contribute thus to a very low porosity of the sintered material [10]. 

The powder metallurgical route was used for the processing of various Al-based alloys, including the Al7075 alloy or its modifications. The combination of gas atomization and high pressure cold deposition was used for the preparation of a compact Al7075 alloy [11]. The main problem of these compacts was their inhomogeneity along the direction of deposition. The Al7075 prepared using spray-forming followed by extrusion exhibited extremely high porosity (up to 20 vol %) [12]. A combination of gas atomization and semi-solid rolling was tested at the Al-Zn-Mg-Cu-Zr alloy [13]. The Al7075 alloy modified by the addition of Zr was processed by extrusion of the atomized powders; however the fine-grained microstructure of atomized powder particles was completely changed, and elongated grains with numerous low angle boundaries (typical for hot extruded materials) were formed [14,15]. The Al7075 compacts prepared by a combination of gas atomization and spark plasma sintering retained the original microstructure of the gas atomized material and no significant grain growth was observed [16,17]. A negligible porosity was observed in these compacts. 

A positive influence of milling on the microstructure refinement was observed in the Al-Zn-Mg-Cu-Zr alloy prepared by a combination of mechanical alloying (milling of elemental powders) and hot pressing [18]. However, hot pressing was not enough efficient in the consolidation of the material and 3–7 vol % of pores were observed. Nanocrystalline grains were detected in a mechanically milled and hot pressed Al7075 alloy [19]. The influence of milling parameters (speed, temperature and time of milling) on the microstructure and microhardness of the Al7075 alloy was tested in [20]. The formation of a bimodal grain size distribution was typical for these samples. Recently, a sub-microcrystalline microstructure with a porosity below 1 vol % was observed in the Al7075 alloy modified by the addition of Zr, which was milled in a planetary mill and then spark plasma sintered [21]. 

Similar processing route as in [21] was used for the processing of the Al7075 alloy in the present research. Our work has focused on the following topics:

- to investigate the microstructure of the gas atomized and milled powder particles, especially the distribution of intermetallic phases, grain size, and the type of interfaces
- to investigate the influence of SPS on the microstructure and phase composition
- to investigate the microhardness of both powder and SPS materials
- to investigate the microstructural stability of the compact material at elevated temperature.

## 2. Materials and Methods

The chemical composition of the investigated gas atomized Al7075 powder, stated by X-ray fluorescence (XRF) is listed in Table 1.

Al7075 alloy powder was prepared by nitrogen gas atomization by Nanoval GmbH & Co. KG, Berlin, Germany. The powder was sieved down to 50 µm, the resulting average particle size, stated by the producer, was 22.6 µm. Mechanical milling of gas atomized powder was performed in a planetary ball mill Retsch PM 100 CM under inert Ar atmosphere at room temperature. The powder was milled in a steel jar using steel balls for 8 h with 400 revolutions per minute (RPM). The ball-to-powder mass ratio was 40:1. The gas atomized and milled powders are further referred as Al7075_A and Al7075_M, respectively. Both atomized and milled powders were sintered in an FCT SPS-HP25 sintering device at 425 °C under 80 MPa for 2 min using a graphite tooling. At the beginning of the sintering 5 MPa initial pressure was applied on the powder. Maintaining the pressure, the samples were free heated up to 400 °C with a heating rate of 80 °C/min, the sintering temperature (425 °C) was reached with a heating rate of 25 °C/min. Simultaneously with temperature, the uniaxial pressure was increased up to 80 MPa. After the 2 min holding time, the sample was unloaded and free-cooled. SPS produced cylindrical compacts with a diameter of 20 mm and height of 5 mm. The SPS compacts prepared from gas atomized and milled powders are further referred to as Al7075_AC and Al7075_MC, respectively. The high temperature stability of the samples was tested by annealing at 425 °C for 1 h followed by water quenching. The heat treated samples are further denoted as Al7075_AC_HT and Al7075_MC_HT.

The powders morphology was investigated by light microscopy (LM) using an Olympus GX51 (Olympus, Prague, Czech Republic) microscope. For this investigation, the powder particles were cold-mounted in an acrylic cold mounting resin and then metallographically processed. Scanning electron microscopy (SEM) was utilized to study the microstructure in backscattered electron (BSE) imaging mode, further to investigate material’s grain structure by electron back-scatter diffraction (EBSD), and to study the chemical composition using energy dispersive X-ray spectroscopy (EDS). These measurements were carried out in FEI Quanta 200F scanning electron microscope (FEI, Brno, Czech Republic). Powder material was hot mounted (180 °C, 2.5 min) by a conductive resin with carbon filler. The investigation of compact samples was performed on thin plates cut parallel to the direction of applied pressure in SPS. All samples were metallographically ground and polished. Samples for EBSD were ion polished in Leica EM RES102.

The finest microstructural details were investigated using transmission/scanning transmission electron microscopy (TEM/STEM) using a JEOL 2200FS microscope (JEOL, Peabody, MA, USA) operating at an accelerating voltage of 200 kV. Lamella from the milled powder was prepared by focused ion beam (FIB) milling in a Zeiss Auriga Compact SEM and mounted onto a copper TEM grid. From SPS compacts, discs with a diameter of 3 mm were cut from a plane parallel to the direction of load applied during SPS. Thin foils for TEM observations were prepared by Gatan 656 dimple grinder (Gatan, Pleasanton, CA, USA) and Leica EM RES102 ion polisher (Leica Microsystems, Wetzlar, Germany).

Microhardness measurements were performed using a microhardness tester Quanta Q10A+ (Qness GmbH, Golling, Austria) equipped with a Vickers indenter. For powder material, 15 different cold mounted powder particles were measured with a load of 5 g. The microhardness of compacts was measured with a load of 50 g from at least 20 indentations.

The X-ray diffraction (XRD) measurements were carried out on a vertical θ-θ diffractometer D8 Discover (Bruker AXS, Brno, Czech Republic) equipped with a divergent beam optics using CuKα radiation. The diffracted beam was detected by a 1D detector LynxEye. Phase identification was done using Diffrac. Eva with accessed PDF-2 database of crystalline phases. Quantitative Rietveld refinement was performed in TOPAS V5 (Bruker AXS, Brno, Czech Republic).

## 3. Results

**Figure 1:**
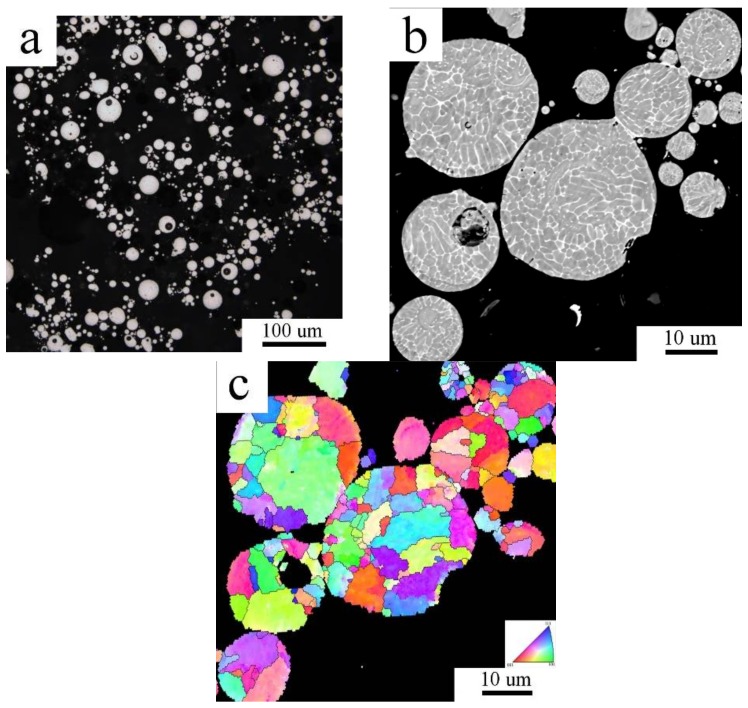
The microstructure of the Al7075_A powder: (**a**) morphology (light microscopy (LM)); (**b**) internal microstructure in backscattered electron (BSE) contrast; (**c**) electron back-scatter diffraction (EBSD) micrograph.

Gas atomization resulted in mainly spherical powder particles (Figure 1a). Figure 1b shows that the microstructure of gas atomized powders is mainly cellular, in some powder particles also columnar or cellular-like dendritic. Only the smallest powder particles exhibit a segregation free solidification microstructure. The EBSD method revealed microstructural heterogeneity of gas atomized powders. The smallest powder particles were found to be single crystalline, whereas larger particles contained several grains of various sizes (Figure 1c). The average grain size of the gas atomized powder was determined to be around 6 µm.

**Figure 2:**
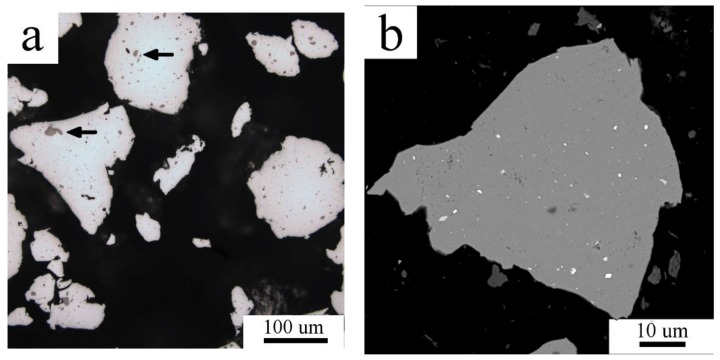
The microstructure of the Al7075_M powder particles: (**a**) morphology (LM); (**b**) internal microstructure in BSE contrast, scanning electron microscopy (SEM).

Mechanical milling in a planetary ball mill highly altered both the powder’s morphology and the internal microstructure. The repeated fracture and cold welding of powder particles during milling resulted in a particle size increase in comparison with the Al7075_A powder (Figure 2a). The continuous layers of intermetallic phases present in the intercellular region of gas atomized powders were destroyed (Figure 2b). A small fraction of Ni and/or Ti impurity particles was found to be introduced into the milled powder particles (see arrows in Figure 2a). The concentration of Ni and Ti in the milled powder was stated by XRF as 1.8 wt % Ni and 1.9 wt % Ti. This contamination stems from the milling jar as a small residue from the last preceding milling process but further experiments revealed that it did not influence the properties of the material. Due to the fine internal microstructure of the milled powders, EBSD was not giving a reasonable orientation image map and the grain size could not be evaluated by this method.

**Figure 3:**
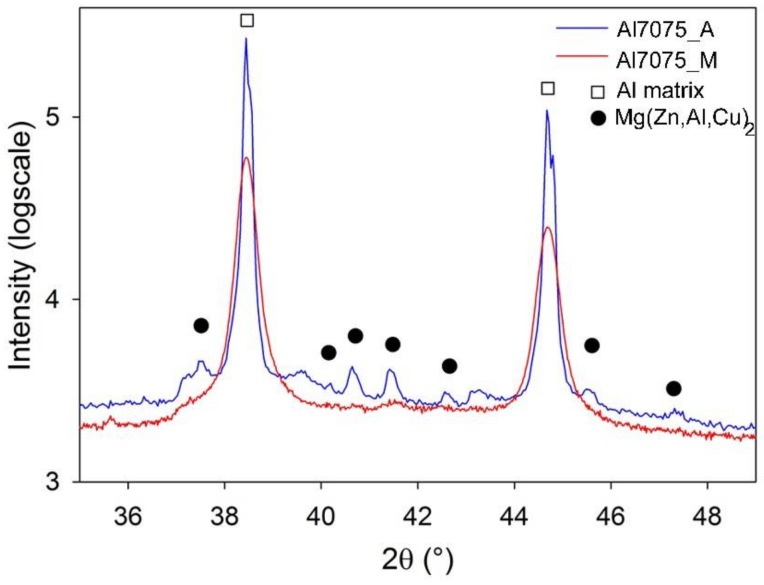
The X-ray diffraction (XRD) pattern of the Al7075_A and Al7075_M powders.

The phase composition of the Al7075_A and Al7075_M powders was estimated using XRD (Figure 3). The Al7075_A powder contains predominantly the Mg(Zn,Al,Cu)_2_ phase (about 1.3 ± 0.2 wt %) in the Al matrix. Nevertheless, peaks corresponding to other phases, usually reported for gas atomized Al7075 alloy could be hidden in the background. The diffraction peaks of the Mg(Zn,Al,Cu)_2_ phase in the Al7075_M powder are much lower but significantly broader. This might be caused by a much finer size of the Mg(Zn,Al,Cu)_2_ phase particles. Despite the observable difference in the diffraction patterns, the rough estimate of the volume fraction of the Mg(Zn,Al,Cu)_2_ phase (1.2 ± 0.1 wt %) is very close to the Al7075_A powder. However, it is necessary to take into account that the volume fractions measured in both powders are at the experimental limit of the method used. The analysis of XRD peak broadening of Al peaks yielded an estimate of the crystallite size of the matrix around 90 nm in the milled powder. No peaks of the Ni- and/or Ti-containing particles were observed which shows a very low fraction of these phases in the Al7075_M powder particles.

**Figure 4:**
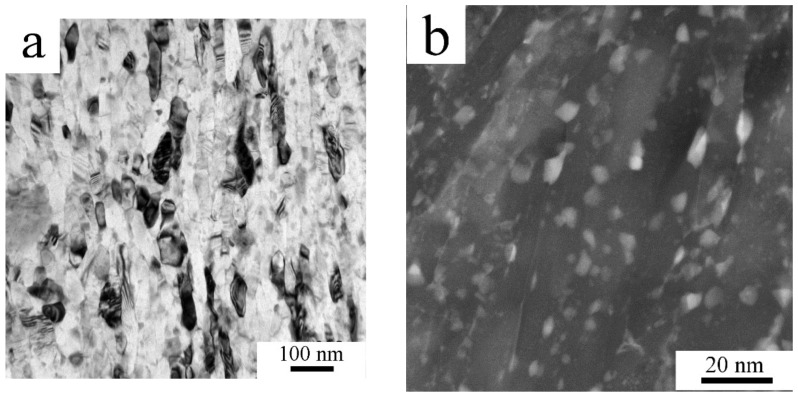
The microstructure of the milled powder, Al7075_M: (**a**) elongated grains, transmission electron microscopy (TEM) and (**b**) nanometer-sized second phase particles, STEM-HAADF.

Finer microstructural details and information about the intermetallic phases present in the Al7075_M powder were obtained by TEM/STEM. Figure 4a shows elongated grains with a size of 50-200 nm. Intermetallic phases—according to TEM-EDS rich in Mg and Zn—were found situated predominantly along the boundaries, as presented on a high angle angular dark field (HAADF) image in Figure 4b.

**Figure 5:**
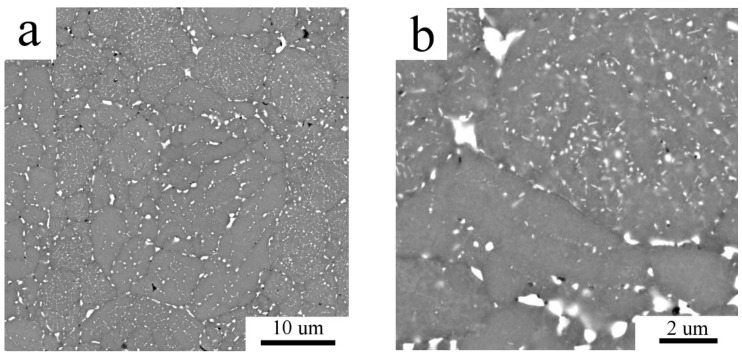
The microstructure of the Al7075_AC compact: (**a**) BSE contrast image; (**b**) detail of the BSE contrast image, SEM.

**Figure 6:**
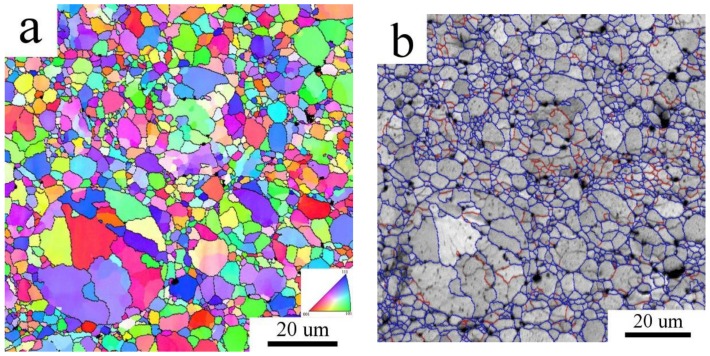
The EBSD micrograph of the Al7075_AC compact, (**a**) inverse pole figures; (**b**) distribution of grain boundaries, boundaries with misorientation angles above 15° in blue, boundaries between 5° and 15° in red.

SPS of both Al7075_A and Al7075_M powders led to compacts with a very low porosity (below 1 vol % and 0.4 vol %, respectively). The microstructure of the Al7075_AC compact is shown in Figure 5. The continuous layers of intermetallic phases present in the atomized powder were broken during SPS and a chain-like microstructure along cell boundaries was formed (Figure 5a). The detail of the SEM-BSE image (Figure 5b) shows that the chains in the interior of the former atomized powder particles are formed by smaller second phase particles with a size of a few hundred nm. Additionally, coarser second phase particles with the size up to 1 µm were observed at the surfaces of original powder particles. The inhomogeneity of the grain size observed in the Al7075_A powder is retained also in the Al7075_AC compact (Figure 6a). The average grain size of the Al7075_AC compact was determined to be 5.2 ± 1.7 µm. The high standard deviation reflects the grain size heterogeneity mentioned above. The distribution of grain boundaries (Figure 6b) shows the prevailing high-angle character of the interfaces present in the Al7075_AC compact.

**Figure 7:**
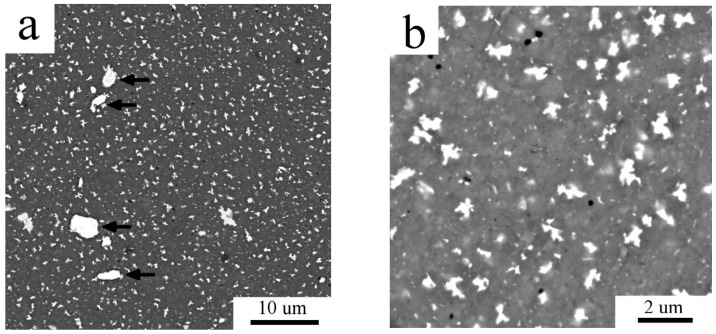
The microstructure of the Al7075_MC compact. (**a**) BSE contrast image; (**b**) detail of the BSE contrast image, SEM.

The microstructure of the Al7075_MC compact is documented in Figure 7a,b. Beside the Ni and Ti impurity particles denoted by arrows in Figure 7a, there are second phase particles of two size scales (Figure 7b). The larger, irregularly shaped precipitates with the size up to 1 µm are homogeneously distributed and, according to EDS measurement, these particles are rich in Zn and Mg. Simultaneously, numerous homogeneously distributed finer second phase particles, spherical and elongated, with the size below 200 nm are present.

**Figure 8:**
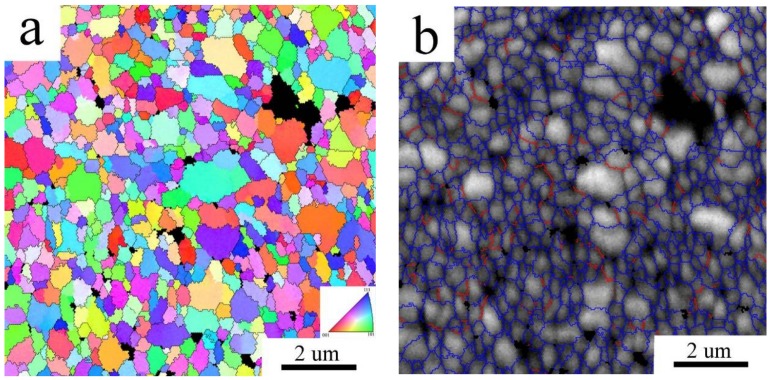
The EBSD micrograph of the Al7075_MC compact, (**a**) inverse pole figures; (**b**) distribution of grain boundaries, boundaries with misorientation angles above 15° in blue, boundaries between 5° and 15° in red.

The grain size of the Al7075_MC compact was evaluated from the EBSD micrographs to be 0.55 ± 0.24 µm. This grain size is about one order finer than that observed in the Al7075_AC compact and the microstructure is more homogeneous (Figure 8a). The distribution of grain boundaries confirms the prevailing high angle character of interfaces in the Al7075_MC compact (Figure 8b).

**Figure 9:**
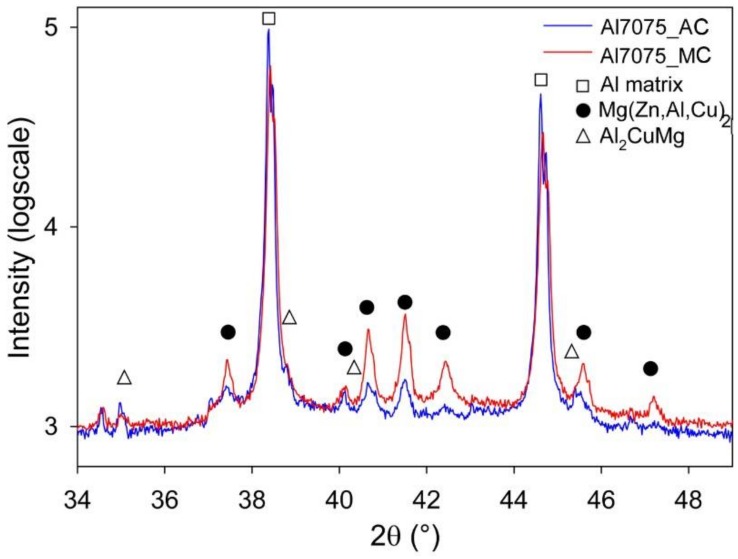
The XRD patterns of the Al7075_AC and Al7075_MC compacts.

SPS at 425 °C influenced also the materials phase composition (Figure 9). The content of the Mg(Zn,Al,Cu)_2_ phase in the Al7075_AC compact (1.2 wt %) was nearly the same as that of the Al7075_A powder (1.3 wt %), moreover, 0.9 wt % of the Al_2_CuMg phase was detected in the Al7075_AC compact. In the case of the Al7075_MC compact, even more remarkable increase in the content of the Mg(Zn,Al,Cu)_2_ and Al_2_CuMg phases was observed (up to 5.6 wt % and 1.2 wt %, respectively).

**Figure 10:**
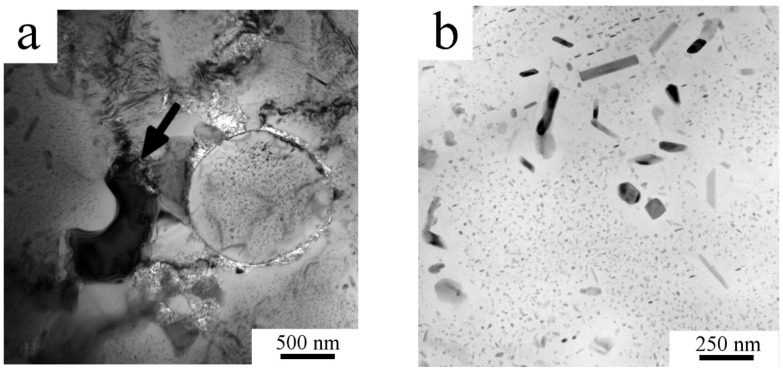
The microstructure of the Al7075_AC compact (**a**) and its detail (**b**), TEM.

A more detailed insight into the compacts’ microstructure and phase composition was obtained using TEM. Figure 10a shows TEM micrograph of Al7075_AC sample. Large precipitates with a size up to 1 µm were found between the former powder particles (see the arrow in Figure 10a). These particles were identified with a selected area electron diffraction (SAED) as MgZn_2_. Moreover, a dense distribution of small nm sized precipitates was detected. A detail of this microstructure is presented in Figure 10b. The second phase particles with a size of several tens of nm to hundreds of nm are a part of the chain-like arrangement observed already by SEM. The larger second phase particles are among small nm sized particles. Depleted zones in the vicinity of the coarser precipitates are clearly visible (Figure 10b). 

**Figure 11:**
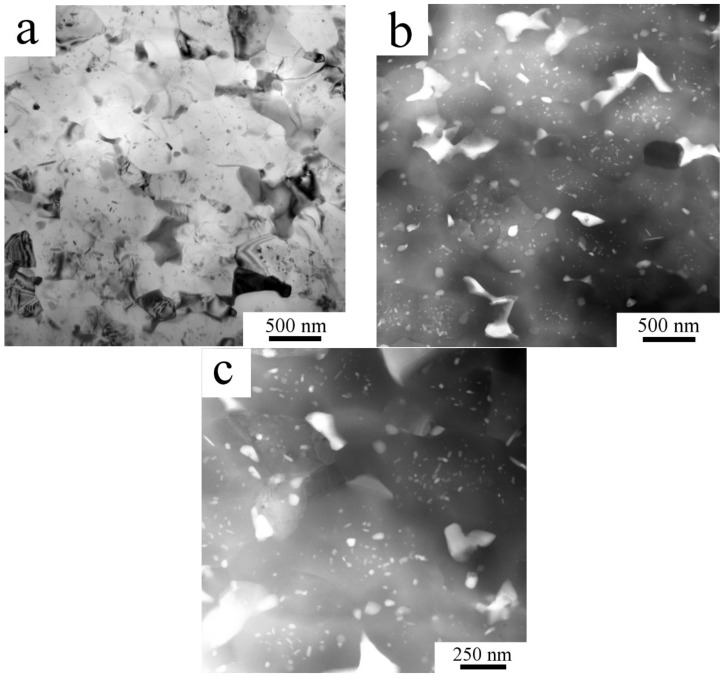
The microstructure of the Al7075_MC compact: (**a**) grain structure, STEM-BF; (**b**) particles; (**c**) detail of particles, STEM-HAADF.

Due to the much finer microstructure of the Al7075_MC compact, its microstructural details were studied by STEM. Figure 11a shows the grain structure and larger irregularly shaped second phase particles located predominantly at triple points or along grain boundaries. The typical size of these precipitates is below 500 nm. Simultaneously, much smaller precipitates (spherical or elongated) of the size below 50 nm are present predominantly in the grain interiors (Figure 11b). A detail in the Figure 11c shows depleted zones along grain boundaries.

The changes in microstructure and phase composition occurring during milling and sintering influence the strength of studied materials, which was characterized by Vickers microhardness. The HV values measured in both powder and compact materials are summarized in Table 2. A large microhardness increase was observed after milling. Whereas SPS of originally gas atomized powder resulted in a slight microhardness increase, a large drop was observed in the Al7075_MC compact.

**Figure 12:**
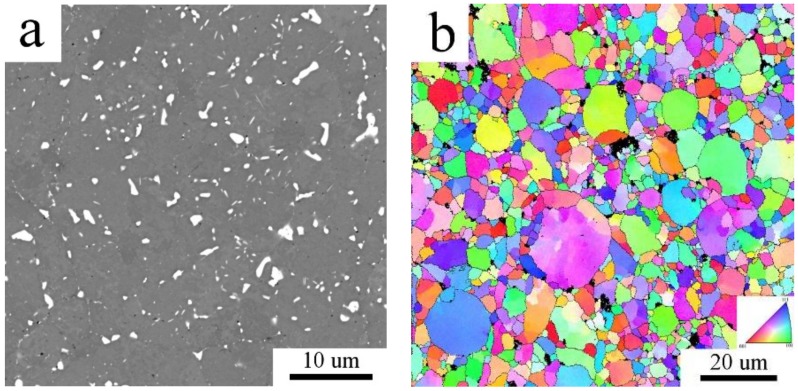

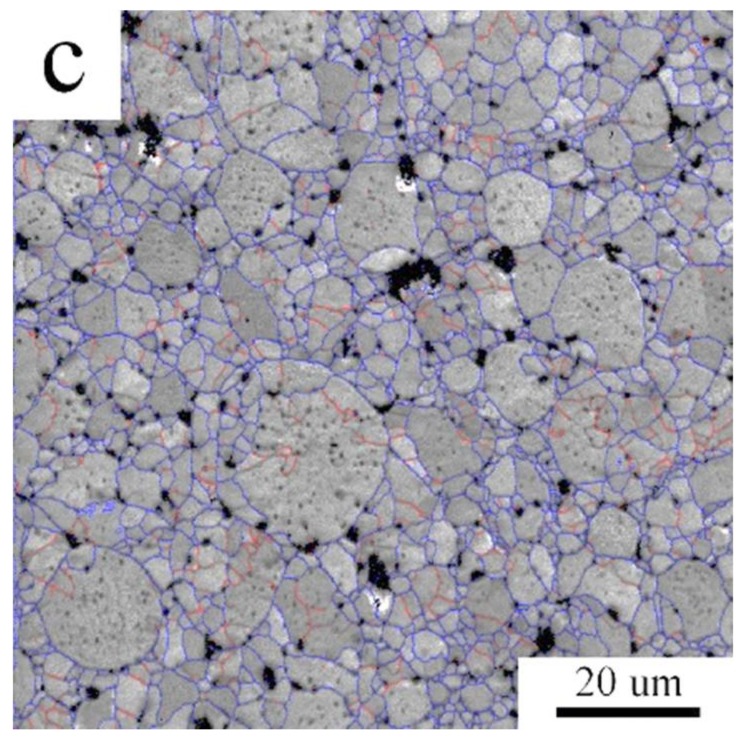
The microstructure of the Al7075_AC_HT compact (annealed 425 °C/1 h): (**a**) BSE contrast image; (**b**) EBSD inverse pole figure; (**c**) distribution of grain boundaries, boundaries with misorientation angles above 15° in blue, boundaries between 5° and 15° in red.

**Figure 13:**
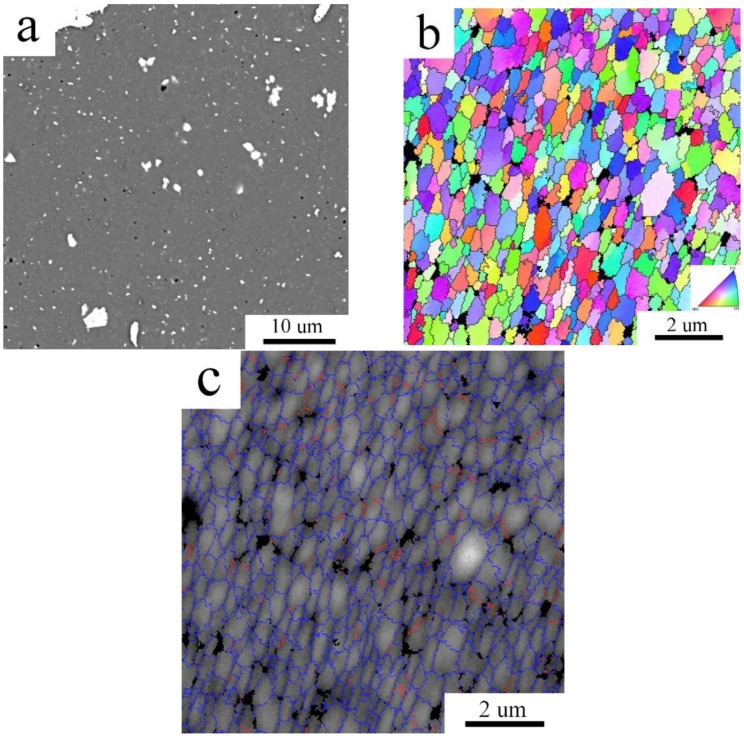
The microstructure of the Al7075_MC_HT compact (annealed 425 °C/1 h): (**a**) BSE contrast image; (**b**) EBSD inverse pole figure; (**c**) distribution of grain boundaries, boundaries with misorientation angles above 15° in blue, boundaries between 5° and 15° in red.

**Figure 14:**
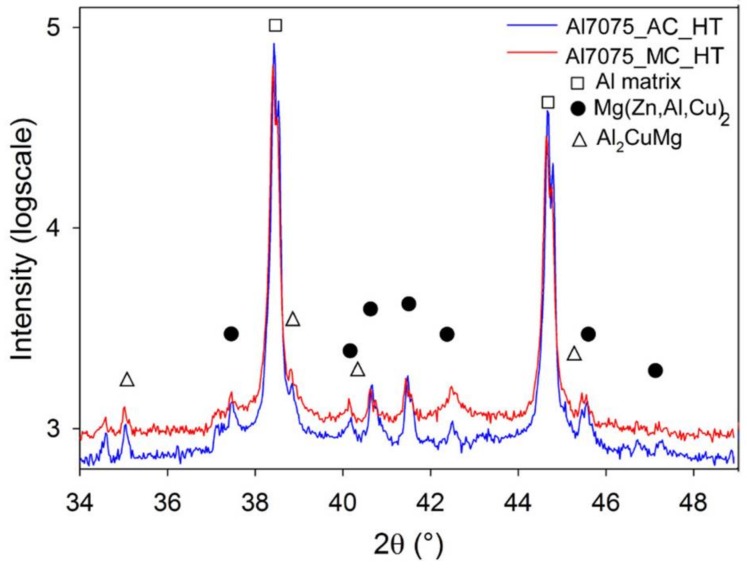
The XRD patterns of the annealed Al7075_AC_HT and Al7075_MC_HT compacts.

Beside the effect of SPS on the materials microstructure and microhardness, the high temperature stability of the resulting microstructure was investigated too. Figure 12 and Figure 13 document the microstructure of the samples heat treated at 425 °C for 1h followed by water quenching, Al7075_AC_HT and Al7075_MC_HT. SEM revealed a coarsening of some second phase particles at the expense of others in both samples (compare to Figure 5 and Figure 7). XRD measurement (Figure 14) documented that high temperature annealing led to precipitation of Al_2_CuMg phase in both compacts, whereas it caused a partial dissolution of the Mg(Zn,Cu,Al)_2_ phase (see Table 3).

**Figure 15:**
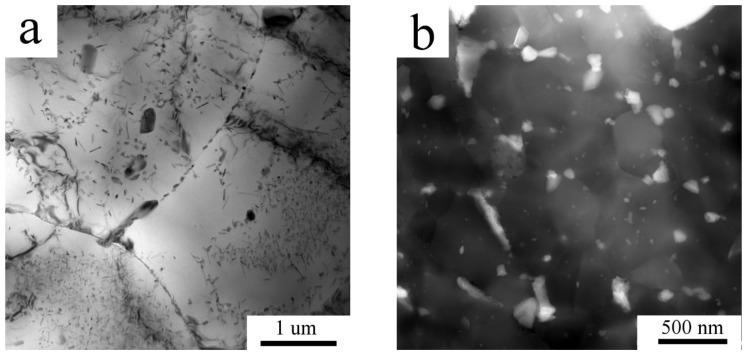
The microstructure of the compacts annealed 425 °C/1 h: (**a**) Al7075_AC_HT, TEM; (**b**) Al7075_MC_HT, STEM.

TEM investigations showed fine microstructural details of the heat treated samples. The Al7075_AC_HT compact was found to exhibit large precipitation free zones around largest precipitates and powder particle boundaries after annealing (Figure 15a). The Al7075_MC_HT compact showed a remarkably lower content of small second phase particles inside the grains (Figure 15b), when compared with the non-heat treated microstructure. 

In contrast to the changes in phase composition, the compacts grain size was found not to be altered by annealing (Figure 12b and Figure 13b). The values of average grain sizes of the non-annealed and annealed samples are equal within the measurements standard deviation (Table 4). High temperature heat treatment altered also the samples microhardness (Table 4). It caused a drop of microhardness in Al7075_AC sample, whereas the microhardness of Al7075_MC sample was found to be increased due the heat treatment. 

## 4. Discussion

It is well known that the solidification morphology of gas atomized powder particles depends generally on their size, i.e., on the solidification rate. Gupta [22] reported a segregation free microstructure of the nitrogen atomized Al-Ti alloy only in droplets with a diameter below 10 μm, cellular morphology was found in droplets between 10 and 100 mm, and dendritic morphology in larger droplets. Similar results were published in [23] for the Al-4.5 wt % alloy and in [11] for the Al7075 alloy. A combination of prevailing cellular and less frequent dendritic-like morphology was observed in our previous investigation of the nitrogen atomized Al7075 powder where about 90% of the powder material was below 70 μm [16,17]. The average droplet size of the material studied in the present research was finer −22.6 μm. As expected, the microstructure was mainly cellular and the cells with the mean size of 6 μm were separated predominantly by high angle boundaries. The smallest droplets were even single crystalline with a featureless microstructure. The intercellular regions contained especially the Mg(Zn,Al,Cu)_2_ phase. 

Mechanical milling completely destroyed the cellular morphology formed during solidification of atomized droplets (Figure 2). The XRD investigation revealed nearly negligible diffraction peaks corresponding to the Mg(Zn,Al,Cu)_2_ phase. However, a proper analysis of XRD patterns proved the presence of this phase. The corresponding diffraction peaks are very broad which can be caused predominantly by a very small size of precipitates and also by their lower ordering resulting from severe plastic deformation during milling. For example, Lukáč et al. [24] estimated the size of coherently diffracting domains of the second phase to be close to 100 nm in a cryomilled Al7075 alloy. In the present study, direct observation of the Al7075_M powder using STEM revealed even much finer precipitates with the size close to 10 nm (Figure 4b). These precipitates might be too small to be detected in XRD experiments and the fraction of second phase particles determined by XRD might be thus underestimated. Figure 2 shows a contamination of the milled powder by Ti- and Ni-rich particles of the size close to 1 μm coming from the last preceding milling process. These relatively large particles are stable during the following processing of the material and do not influence its microstructure development. 

Literature data document that mechanical milling is a very efficient technique for processing of nanocrystalline materials. Milling of the Al7075 alloy at room temperature for 20 h resulted in a grain size of 26 nm [19], cryo-milling for 10 h in 28 nm [25]. Elongated grains with a typical size of 20 × 100 nm were observed in the Al7075 alloy doped by Zr in [21]. Slightly coarser elongated grains (typically 50 × 200 nm) were observed using TEM in the present material milled at room temperature for only 8 h (Figure 4a). This very small grain size is close to the spatial resolution limit for EBSD and this caused that no reasonable information on the grain size or character of grain boundaries was obtained from this method.

Grain refinement, precipitate refinement, and deformation energy introduced into the gas atomized powder during milling enhance significantly microhardness values (from 127 to 327 HV). The value measured in the milled powder is nearly twofold of that observed in a peak aged Al7075 alloy prepared using ingot metallurgical processing (170 HV) [26] or in the melt-spun ribbons of the Al-Zn-Mg-Cu-Zr alloy after optimized thermal treatment (186 HV) [27]. It is also much higher than values found in Al7075 powders milled in laboratory attritor both at room temperature for 8 h (229 HV) or at cryo-temperature for 3 h (220 HV) [20]. On the other side, a comparable microhardness value (343 HV) was measured in the Al7075 + Zr alloy milled in the same planetary mill as in the present investigation [21]. The comparison of these results suggest that a proper choice of the mill device can influence the microstructure and mechanical properties of the milled product in a more significant manner than a choice of milling conditions (temperature, RPM). The planetary milling device gives better results in shorter milling times. 

There are many investigations on Al-based materials showing that spark plasma sintering is a very efficient technique for preparation of bulk materials with a very low porosity [28,29,30]. The sintering temperature and applied load are the most important parameters influencing both the resulting porosity and the microstructure of the sintered compact. An increasing sintering temperature contributes to a better consolidation of the powder material, however, it can also result in grain coarsening, i.e., destroy the main benefit gained during preceding rapid solidification or mechanical milling. The sintering parameters (500 °C, 75 MPa, 3 min) used in our previous investigation of the gas atomized Al7075 alloy with a coarser initial size of droplets [17] led to a 99.6% density but the size of some grains in the SPS compact was above 20 μm. Much finer grain size and very low porosity (deeply below 1 vol. %) were found in SPS compacts of the Al7075 + 1 wt % Zr alloy sintered at 425 °C, 80 MPa and holding time 4 min [21]. In the present investigation, the same sintering temperature and load were used and the holding time was shortened to 2 min. The porosity of compacts prepared both from gas atomized and milled powders was retained below 1 vol. %. 

The Al7075 alloy is an age-hardenable alloy whose phase composition is strongly dependent on its thermal history. The volume fraction of second phases is relatively low both in the atomized and milled powders, especially as a consequence of rapid solidification. During SPS, the powders were exposed to elevated temperatures, which could alter their phase composition. The temperature of SPS is about 50 °C below the solution temperature for this alloy. Considering a short time spent during SPS at this temperature, no massive dissolution of existing second phase particles can be expected. A partial decomposition of the supersaturated powder matrix and formation of small precipitates can occur during heating to the sintering temperature and especially during a free cooling of the SPS compacts from the SPS temperature. 

A relatively small increase in the fraction of second phases was detected by XRD in the Al7075_AC compact and SEM observations revealed a change in their distribution. The continuous layers of non-matrix phases located at intercellular boundaries were partially dissolved and replaced by individual precipitates of the sub-micrometer size. Due to a short time spent at elevated temperatures the solute atoms could not diffuse over a long distance and new precipitates are formed predominantly at the same places. A faster diffusion along the surface of original powder particles (probably due to a higher temperature during SPS at contact points) resulted into formation of much coarser precipitates (with the size close to 1 μm) at boundaries of original powder particles. Very small precipitates (with the size of the order of 10 nm) observed by TEM and STEM are probably formed by decomposition of the supersaturated matrix phase. 

A remarkable increase in the fraction of second phases was detected by XRD experiment in the Al7075_MC compact. This is in agreement with SEM observations showing much higher number of precipitates of the size close to 1 μm (compare Figure 2b and Figure 7a). It can be assumed that a faster diffusion of solutes due to increased amount of lattice defects (grain boundaries, dislocations) in the milled nanocrystalline powder is a reason for a faster precipitation. In comparison with the Al7075_AC compact, these relatively coarse precipitates are more homogeneously distributed. The comparison of Figure 10b and Figure 11c suggests a lower fraction of the finest precipitates in the Al7075_MC compact. Observed changes in the phase composition are very similar to those observed in the SPS Al7075 + 1 wt % Zr compacts prepared using similar processing parameters [21].

EBSD investigation revealed that the grain size observed in the gas atomized powder did not significantly change during SPS. Some original powder particles are still visible in the Al7075_AC compact, the structure is heterogeneous, and the grain size corresponds to the grain size of the Al7075_A powder. It can be supposed that second phases, either in the form of continuous layers or individual particles, are located along cell boundaries and stabilize their size. A completely different situation was observed in the milled material. The deformation energy stored in powder particles during their mechanical milling represents a driving force for a remarkable coarsening of the original nanocrystalline structure during SPS. A relatively homogeneous recrystallized sub-microcrystalline structure with the mean grain size of 550 nm and containing mostly high-angle grain boundaries is formed in the Al7075_MC compact. Particles of non-matrix phases located at triple points retard further grain coarsening. Despite this coarsening, the grain size of the Al7075_MC compact remains in the sub-micrometer range and is much finer and especially much more homogeneous than that observed previously in SPS compacts prepared from powders milled in attritor [20]. It is also more than one order of magnitude finer in comparison with ingot metallurgical Al7075 alloy subjected to a special recrystallization treatment [2,31] and comparable with that observed for the same alloy after ECAP [4,5]. 

**Figure 16:**
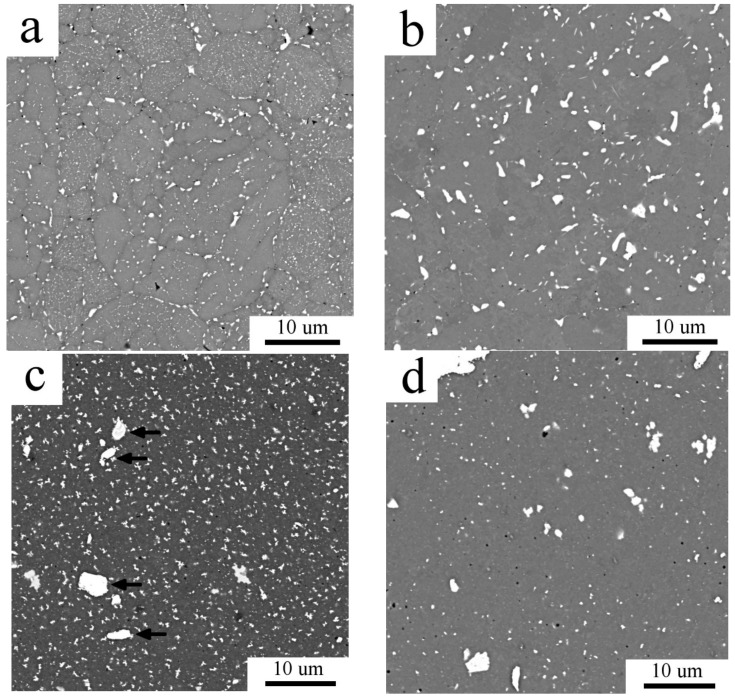
The microstructure of SPS compacts in BSE contrast: (**a**) Al7075_AC; (**b**) Al7075_AC_HT; (**c**) Al7075_MC; (**d**) Al7075_MC_HT, SEM.

As mentioned in the introduction, poor high temperature stability is a serious problem in utilization of Al-based alloys. Recrystallization, grain growth, and coarsening of strengthening phases reduce significantly their strength. An abnormal grain growth was observed e.g., in the sub-microcrystalline Al7075 prepared using ECAP already at 300 °C [4,5]. The stability of both our SPS compacts was tested by annealing at a relatively high temperature of 425 °C. The main result of this experiment is that the grain size remains nearly unaffected which is of great importance especially in the sub-microcrystalline Al7075_MC compact. Changes in the phase composition of both compacts occurring due the annealing are documented in Figure 16, which compares the micrographs from Figure 5 and Figure 12 for the Al7075_AC compact, and from Figure 7 and Figure 13 for the Al7075_MC compact. Whereas some coarsening of second phase particles was observed in the Al7075_AC compact, a partial dissolution of coarser second phase particles (in agreement with the XRD experiment) is typical for the Al7075_MC compact.

The changes in microstructure and phase composition occurring during SPS and following thermal treatment influenced the microhardness values. It was not the aim of this research to perform any quantitative analysis of strengthening, however, four strengthening mechanisms have to be considered for a qualitative analysis—strengthening by second phase particles, by solution atoms, by grain boundaries, and by dislocations. All these mechanisms are expected to contribute to extraordinary high microhardness values in the milled Al7075_M powder. Grain coarsening, release of stored deformation energy, and precipitation of relatively coarse second phase particles can explain the deterioration of microhardness in the Al7075_MC compact. On the other side, precipitation of very small second phase particles inside grains can be responsible for an increase in microhardness in the Al7075_AC compact (as compared with the Al7075_A powder). Coarsening of both coarser precipitates (see Figure 16a,b) and very fine precipitates inside grains (compare Figure 10b and Figure 15a) in the Al7075_AC_HT compact results in a decrease of microhardness during annealing at 425 °C. On the other side, the Al7075_MC_HT compact contains finer second phase particles and probably also a higher amount of solutes in matrix which increases its microhardness. The value of 178 HV is slightly above that for the peak aged alloy prepared through ingot metallurgy, despite the fact that no special thermal treatment was applied, and despite the exhibition of the Al7075_MC_HT compact to the high temperature of 425 °C.

## 5. Conclusions

1. Mechanical milling of the gas atomized powder of the Al7075 alloy in a planetary mill device refines the grain size to a nanometer range. Simultaneously the cellular solidification structure of the gas atomized Al7075_A powder is completely destroyed and very small second phase particles are observed, especially along grain boundaries. The milled Al7075_M powder exhibits an extraordinary high microhardness of 327 HV as a result of small grain size, large stored deformation energy, and very small size of second phase particles.
2. Compacts with a negligible porosity (below 1 vol. %) are prepared using SPS from both gas atomized and milled powders. Despite a relatively low temperature and short time of sintering, SPS influences the microstructure and phase composition of both materials.
3. In the compact prepared from gas atomized powder, the grain size is retained during SPS. The continuous layers of intermetallic phases are partially destroyed and replaced by individual particles located in the same places. Additionally, precipitation of nanometer-sized particles occurs inside grains, which contributes to a microhardness increase.
4. In the compact prepared from milled powder, grain coarsening to the sub-microcrystalline range occurs. Further, a massive precipitation of second phase particles takes place. The coarser particles with the size below 1 μm are located mostly at triple points. The density of fine precipitates inside grains is slightly lower than in the compact sintered from atomized powder. The microstructural changes occurring during SPS significantly reduces the microhardness values.
5. Annealing of both SPS compacts at 425 °C for 1 h does not alter their grain size, however, it modifies the distribution of second phase particles. Coarsening of these particles, observed in the annealed compact prepared from atomized powder, results in a decrease of microhardness. On the other side, a partial dissolution of the coarsest second phases particles observed in the annealed compact, prepared from milled powder, contributes to a microhardness increment.
6. SPS of the milled Al7075 powder produces a compact material with the grain size in the sub-microcrystalline range which remains stable even during annealing at 425 °C. This annealing results in an increase of microhardness to values exceeding those observed in the peak aged Al7075 alloy prepared using ingot metallurgical route.

## Figures and Tables

**Table 1:** The chemical composition of the investigated gas atomized Al7075 powder.

Element	Weight Percentage (wt %)
Zn	7.00 ± 0.08
Mg	2.40 ± 0.05
Cu	2.19 ± 0.04
Fe	0.12 ± 0.01
Si	0.07 ± 0.01
Al	balance

**Table 2:** The microhardness of both powder and compact materials.

Material	Microhardness (HV)
Al7075_A powder	127 ± 41
Al7075_M powder	327 ± 32
Al7075_AC compact	151 ± 3
Al7075_MC compact	154 ± 7

**Table 3:** The evolution of second phase particle content in the Al7075_AC and Al7075_MC compacts due to annealing at 425 °C for 1 h.

Sample	Mg(Zn,Cu,Al)_2_ (wt %)	Al_2_CuMg (wt %)
Al7075_AC	1.2 ± 0.1	0.9 ± 0.1
Al7075_AC_HT	1.4 ± 0.1	1.8 ± 0.1
Al7075_MC	5.6 ± 0.2	1.2 ± 0.2
Al7075_MC_HT	1.7 ± 0.2	1.4 ± 0.2

**Table 4:** The grain size determined from EBSD images and microhardness values after annealing 425 °C/1 h.

Material	Grain Size (µm)	Microhardness (HV)
Al7075_AC	5.2 ± 1.7	151 ± 3
Al7075_AC_HT	6.1 ± 1.8	125 ± 2
Al7075_MC	0.55 ± 0.24	154 ± 7
Al7075_MC_HT	0.57 ± 0.18	178 ± 5
